# Dyscalculia in Early Adulthood: Implications for Numerical Activities of Daily Living

**DOI:** 10.3390/brainsci12030373

**Published:** 2022-03-11

**Authors:** Giulia Vigna, Enrico Ghidoni, Francesca Burgio, Laura Danesin, Damiano Angelini, Silvia Benavides-Varela, Carlo Semenza

**Affiliations:** 1 Faculty of Social and Behavioral Sciences, Leiden University, 2333 AK Leiden, The Netherlands; g.vigna@umail.leidenuniv.nl; 2 Babylab, University of Padova, 35131 Padova, Italy; 3 Clinical Neuropsychology and Adult Dyslexia Unit, Neurology Department, Arcispedale S. Maria Nuova, 42123 Reggio Emilia, Italy; ghidoni.enrico@alice.it (E.G.); damiano.angelini@ausl.re.it (D.A.); 4 IRCCS San Camillo Hospital, 30126 Venezia, Italy; francesca.burgio@hsancamillo.it (F.B.); laura.danesin@hsancamillo.it (L.D.); 5 Department of Developmental Psychology and Socialisation, University of Padova, 35131 Padova, Italy; 6 Department of Neuroscience, University of Padova, 35128 Padova, Italy; 7 Padova Neuroscience Center, University of Padova, 35131 Padova, Italy; carlo.semenza@unipd.it

**Keywords:** developmental dyscalculia, NADL, numerical activities, daily living, lifespan skills

## Abstract

Numerical abilities are fundamental in our society. As a consequence, poor numerical skills might have a great impact on daily living. This study analyzes the extent to which the numerical deficit observed in young adults with Developmental Dyscalculia (DD) impacts their activities of everyday life. For this purpose, 26 adults with DD and 26 healthy controls completed the NADL, a standardized battery that assesses numerical skills in both formal and informal contexts. The results showed that adults with DD had poorer arithmetical skills in both formal and informal settings. In particular, adults with DD presented difficulties in time and measure estimation as well as money usage in real-world numerical tasks. In contrast, everyday tasks regarding distance estimation were preserved. In addition, the assessment revealed that adults with DD were aware of their numerical difficulties, which were often related to emotional problems and negatively impacted their academic and occupational decisions. Our study highlights the need to design innovative interventions and age-appropriate training for adults with DD to support their numerical skills as well as their social and emotional well-being.

## 1. Introduction

The ability to deal with numbers is fundamental in our modern society. From managing money to remembering PIN numbers or codes, numerical skills are continuously needed during an adult’s daily life. People with Developmental Dyscalculia (DD) often have poor mathematical skills that may have an impact also in their daily lives [1]. DD is a specific learning disorder with a reported prevalence in the population of 3–7% [2,3]. According to the fifth edition of the Diagnostic and Statistical Manual of Mental Disorder (DSM-5) edited by the American Psychiatric Association, people with DD have mathematical skills substantially and quantifiably below those expected for the individual’s chronological age in the domain of number sense (processing and understanding quantities), arithmetical facts retrieval, calculation, and mathematical reasoning [4]. These mathematical difficulties have the following characteristics: (1) persist for at least 6 months, despite the provision of specific interventions; (2) appear during school age and can last until adulthood, interfering with the individual’s academic or occupational performance; (3) cannot be explained as a consequence of brain damage or diseases, neurogenetic disorders, premature birth, visual or hearing impairments, intellectual disabilities, or poor psychoeducational stimulation [4].

DD affects the individual throughout the lifespan. The first signs of DD can be usually detected in preschoolers. Common difficulties at this young age involve magnitude processing (enumerating and comparing non-symbolic quantities), counting, and seriation [5]. Later, children with DD show limited access to the mental number line and struggle with transcoding between number words, digits, and quantities. In addition, they might have difficulties in understanding the place–value system, executing oral and written calculations, using arithmetical symbols, memorizing arithmetical facts, solving arithmetical problems [6,7]. Besides the listed domain-specific deficits, children with DD might also show impairments in domain-general abilities, such as working memory, language abilities, attention, and executive functions [8,9]. Furthermore, DD often co-occurs with dyslexia, dysgraphia, dysorthography, attention-deficit/hyperactivity disorder (ADHD), anxiety, depression, and aggressive behaviors [10,11].

While many studies have investigated the impact of DD on children, less is known about DD in adulthood (see [12] for a complete review). Similar to children, adults with DD present both number-specific and domain-general cognitive difficulties [13]. The main domain-specific deficits concern subitizing and enumeration [14,15], number/size interference [16], number bisection [17,18], automatic activation of number magnitude [16], arithmetic fact retrieval [19], arithmetical conceptual understanding [20], alerting and executive attention [17,21], time and duration processing [22]. In addition, deficits in phonological processing, rapid naming, and verbal working memory have also been found [13].

Even if children and adults with DD present similar deficiencies, the implications of this disorder might be different at an older age. While during childhood poor arithmetical skills affect school attainment, mental health, and self-esteem [23], in adulthood, their impact on daily living might concern additional life spheres, such as money usage or job seeking. Some studies have highlighted that people with low numeracy have a lower range of working opportunities, lower salaries, poor financial well-being [24,25,26], and less access to Internet technology (e.g., computers and cell phones) [27]. Furthermore, low numeracy can also have an impact on people’s health, insofar as it distorts perceptions of risks and benefits regarding medical screenings and treatments [28]. However, these findings are mostly derived from cohort studies, national surveys, or reviews that do not distinguish between DD and other causes of low numeracy in the adult population.

To our knowledge, no previous research has provided a complete and specific outline of the impact of DD on adult daily living, taking into account various spheres of life. As recently suggested by Kaufmann et al., (2020), there is a crucial need to elucidate the cognitive and behavioral manifestations of DD in adulthood. Therefore, this study aims to analyze to what extent DD in adulthood affects people’s understating of numbers in their activities of everyday life. It was hypothesized that people with DD should experience greater difficulties in these everyday tasks than healthy control individuals.

## 2. Materials and Methods

### 2.1. Participants

Twenty-six adults with DD (18 females, mean age 25.19 ± 9.19, mean years of education 12.88 ± 2.49) and 26 healthy controls (13 females, mean age 27.61 ± 7.87, mean years of education 13.92 ± 2.78) participated in the study. Participants with DD were consecutively enrolled (from 2013 to 2016) at an Italian clinic specialized in Neuropsychology, Cognitive Impairments and Dyslexia in adulthood (Neuropsicologia Clinica, Disturbi Cognitivi e Dislessia dell’Adulto, IRCCS-Arcispedale S. Maria Nuova, Reggio Emilia) after receiving the diagnosis of DD by a psychologist and an expert neurologist. The diagnosis was made using the Protocollo Test Disturbi Specifici dell’Apprendimento [Specific Learning Disorder Test Protocol] [29]. None of the participants with DD received any treatment during the time of the study, but some of them had benefited from educational, logopedic, or psychological interventions during childhood (for further details, please see the Supplementary Materials, Spreadsheet S3). Comorbidity with other learning disabilities was not an exclusion criterion for DD patients. Specific information about participants’ comorbidities can be found in the Supplementary Materials, Spreadsheet S3. Healthy controls were recruited through media announcements and personal contacts of the authors. They completed only the NADL test (see more details in the Measures section below). Some of the healthy controls (N = 19) were also enrolled in a previous study, which had different research questions and aims and employed different analyses [30]. Participants were excluded if any of the following criteria were met: (1) abnormal vision or hearing that could not be corrected to normal; (2) history of a psychiatric disorder or drugs or alcohol abuse; (3) presence of a genetic, neurological, or educational condition that could affect the test performance. In addition, the controls reported no developmental learning disorders or relevant pathologies that could influence their cognitive performance at the time of testing. This study was approved by the Ethics Committee of the IRCCS San Camillo Hospital. All participants took part in the study on a voluntary basis and provided written consent. The demographic characteristics of all participants are reported in Supplementary Materials, Spreadsheet S1.

### 2.2. Measures

Participants’ formal numerical abilities as well as the impact of numerical abilities on participants’ daily life was assessed using the Numerical Activities of Daily Living (NADL) standardized test [31]. The NADL test is a tool that was created to identify impairments in numerical functions that may cause problems in day-to-day and ecologically valid tasks [31]. It has been widely used, for example, with patients with mild cognitive impairment [32] and with neurofibromatosis type 1 [30]. To date, it has never been used with adults with DD. Notably, in this study, NADL subtests were not used as diagnostic tools, but rather as experimental hypothesis-driven tasks, whose scores in DD people were compared against those of a control group (not the normative data). Specifically, three sections of NADL were administered: the Participant Interview, the Informal Test, and the Formal Test.

The Participant Interview assesses the individuals’ awareness of their numerical abilities. It consists of 10 questions about daily activities that involve numbers (e.g., please pay XX; this is your change, could you please check whether it is correct?).

(a) The Informal Test evaluates the participants’ competencies in everyday numerical activities. It consists of real-world tasks/questions regarding numerical knowledge in the domains of Time (e.g., can you tell me for how long we have been doing this interview?), Measure (what would be the amount of pasta in an average portion?), Distance (can you estimate the distance between your home and this hospital?), Communication (could you please tell me your own telephone number?), General semantic numerical knowledge (e.g., do you remember the dates of the last world war?), and Money (e.g., if a shirt normally costs 50 euros but it is discounted by 10%, how much would you have to pay for it?).

In the NADL interview and NADL informal, there were no texts to read, and all the instructions and answers were orally provided.

(b) The Formal Test assesses the participants numerical abilities acquired at school. It focuses on:
  - Number comprehension, i.e., the ability to match numbers to their corresponding magnitudes. It consists of three subtasks: (1) Number Line Marking (estimating the position of a number on a line whose the extremities are known; the extremities varied between 0 and 10, 0 and 100, and 0 and 1000), (2) Numerosity Comparison (compare the number of squares displayed simultaneously in two sets; quantities varied between 2 and 9), and (3) Digit Comprehension (pointing at the symbolic number that corresponds to the number of squares in a set; the listed numbers were from 1 to 10).
  - Transcoding, i.e., the ability to relate number words to their Arabic symbols. It consists of two subtasks: (1) Reading numbers aloud (the presented numbers were 12, 53, 104, 2600, 65,300) and (2) Writing Numbers to Dictation (the presented numbers were 2, 51, 307, 2005, 42, 300).
  - Mental calculation of additions (N = 6, single-digit operations), subtractions (N = 3, single-digit operations; and N = 3 two-digit minuend and one-digit subtrahend operations), and multiplications (N = 6 one-digit multiplications).
  - Knowledge of rules and principles of calculation. It consists of three subtasks: (1) Arithmetical Rules (N = 7), (2) Addition Principles (N = 4), and (3) Multiplication Principles (N = 4).
  - Written operations with multi-digit additions (N = 6), subtractions (N = 6), and multiplications (N = 6).

The original standardization of the NADL shows adequate reliability and internal consistency, with Cronbach’s alpha = 0.73 [31]. Accordingly, one point is assigned for each correct answer. The maximum total score is 10 for the Participant Interview, 23 for the Informal Test, and 77 for the Formal Test.

### 2.3. Data Analysis

To assess whether the scores in the two groups were normally distributed, we looked at the frequency histograms and performed the Kolmogorov–Smirnov test. Since the data were not normally distributed, non-parametric Wilcoxon rank-sum tests were generally used to compare the results obtained in the NADL test by participants with and without DD. The data of tasks regarding measure, distance, and communication in the informal part of the NADL were categorical, so χ2 tests were used in these cases. Our hypothesis was directional, namely, that people with DD should experience greater difficulties in everyday numerical tasks than healthy control individuals; therefore, all statistical tests were one-sided. In addition, the Holm–Bonferroni correction for multiple comparisons was applied. Finally, to support evidence of absence, a Bayesian analysis was conducted.

## 3. Results

### 3.1. Demographic Differences between the Two Groups

Participants with DD did not significantly differ from controls in sex (χ^2^(1) = 1.997, *p* = 0.158), years of education (U = 288, *p* = 0.353), and age (U = 285, *p* = 0.329).

### 3.2. Numerical Abilities

Table 1 reports a summary of the performance of the two groups on the NADL battery. Raw data supporting these results can be found in Supplementary Materials, Spreadsheet S2.

#### 3.2.1. Participant Interview

Twenty-two participants with DD and twenty-six controls completed the Interview. The results of the Wilcoxon rank-sum tests indicated that individuals with DD scored significantly lower than controls. Moreover, Bayes factors confirmed that there was substantial evidence of H_1_ compared to H_0_ in the Interview scores.

#### 3.2.2. Informal Test (Participants’ Competences in Everyday Numerical Activities)

Twenty-one participants with DD and twenty-six controls completed the Informal Test. Participants with DD scored significantly lower than controls in the Informal Test. In particular, participants with DD scored significantly lower in time estimation, measure, and money usage. Bayes factors confirmed very strong evidence of H_1_ compared to H_0_ in the Total Informal scores and substantial evidence of H_1_ compared to H_0_ in measure and money usage.

On the other hand, no significant difference between DD and controls was found regarding distance estimation, communication, and general semantic numerical knowledge. Bayes factors indicated substantial evidence of H_0_ compared to H_1_ in distance estimation (Table 1).

#### 3.2.3. Formal Test (Participants’ Numerical Abilities Acquired at School)

Twenty-six participants with DD and twenty-six controls completed the Formal Test. Overall, participants with DD scored significantly lower than controls in the Formal Test. This was confirmed by the results of both the Wilcoxon rank-sum and Bayesian tests. There were significant differences between the two groups regarding mental calculation knowledge of arithmetical rules and written calculation, while the two groups did not differ in number comprehension and transcoding. Further details regarding the differences between the two groups in each specific operation within the subdomains of the Formal test can be found in the Supplementary Material, Table S1. Notably, in the present study, the NADL Formal Test was not used as a diagnostic/clinical test of dyscalculia. However, the results of the NADL were highly consistent with those of the diagnostic battery. In particular, we found that the scores for accuracy in arithmetical facts retrieval measured with the diagnostic protocol correlated with single-digit mental multiplications in the NADL (rho = 0.514, *p* = 0.009). In addition, the accuracy in written calculation measured with the diagnostic protocol correlated with the total written calculation in the NADL (rho = 0.589, *p* = 0.002).

## 4. Discussion

This study investigated the extent to which the numerical deficit observed in adults with DD impacts their activities of everyday life. To this end, the performance of adults with DD and healthy controls was compared using a standardized test that assessed a wide range of formal school-level mathematical abilities, as well as the ability to carry out real-world activities regarding numbers.

Firstly, our results show, not surprisingly, that adults with DD have difficulty performing various arithmetical and numerical tasks. In fact, their global accuracy in the Formal section of the NADL was significantly lower than that of the control participants. Significant differences between the groups were evident both in mental and in written calculation tasks, mainly concerning the performance in subtractions and multiplications (see Supplementary Materials, Table S1). Previous studies reported numerical deficits in adults with DD both in arithmetic and in numerosity processing [13,22]. Our results support these findings and extend them to a relatively larger sample of adult participants suffering from DD. Altogether, these studies suggest the presence of a deficit affecting the processing of symbolic mathematical operations. Moreover, our findings confirm that formal numerical deficits in people with developmental dyscalculia do not resolve with age.

Secondly, and most relevant for the purpose of the present work, an impairment in retrieving numerical knowledge and performing calculations applied to ecological situations was also observed. Adults with DD were less accurate than control participants in various informal numerical tasks, including time and measure estimation. Previous studies reported a deficit in the estimation of magnitude and duration in both children [33,34] and adults with DD [22]. Our data, thus, support the hypothesis of a partially shared quantity system across numerical and temporal dimensions [35,36,37]. Moreover, our findings extend previous studies by suggesting that such a duration and measurement processing deficit has a negative impact on the individual’s daily activities, possibly affecting punctuality, resources, and time management (see also the Participants’ structured Interview, below). Notoriously, the perception of distance appeared preserved in our group of DD. In fact, no significant differences were observed compared to the control group in this domain, which was confirmed through Bayesian analyses. These results are in line with previous studies showing a preserved representation of length [22] and space in the population with congenital and persistent number impairment [18]. In addition, our results suggest that spared representations of space also support adequate performance in ecological tasks (i.e., estimations of distance in km) in DD. Altogether, these findings support the view of a deficit of number magnitude representation in DD with a relative preservation of some spatial representations (see also [35]).

This study also revealed that adults with DD were less accurate than control participants in tasks involving simple monetary transactions such as buying a newspaper, calculating change, applying discounts, etc. Despite the many studies of the cognitive and neural basis of DD, to our knowledge, this is the first time the impact of such important disturbances on the daily activities of individuals has been directly measured in a group of adults suffering from DD. Money usage permeates modern societies and is a crucial ability that contributes to independence in many activities of daily living. Assessing financial skills and money usage in clinical settings requires specifically designed tools [38,39]. Unfortunately, the criterion adopted by many practitioners regarding performance in real-world activities is often based on subjective reports of the patient or on generic neuropsychological and school-based mathematical assessments. This procedure is not scientifically justified from either a theoretical or an empirical point of view, not only because the ecological validity of neurocognitive tests is often only moderate [40] but also because clear dissociations between formal mathematical abilities and their use in everyday life have been previously verified. For example, adults with a genetic syndrome—neurofibromatosis I—have been found to be impaired in formal tests of school arithmetic, while they perform like healthy control groups in similar tasks applied to ecological situations [30]. Likewise, various studies have reported that neuropsychological screening tests lead to misclassifications concerning people’s financial inability. For example, in a study by Toffano et al., (2021), a group of older adults who showed low financial ability did not meet the diagnostic criteria for dementia (including MMSE < 24). Vice versa, a group of adults with signs of dementia were able to autonomously deal with financial matters, like healthy older adults [41]. The current study did not find such dissociations using a tool specifically developed to assess real-world activities and money usage. That is, unlike in other pathological populations, ecological contexts do not seem to influence or favor math resolution processes in adults with DD. However, the Money usage subtest only investigates basic financial abilities (i.e., counting currencies and financial estimates); therefore, higher-order skills, such as the ability to recognize fraud attempts, should be assessed with ad hoc tests in future studies.

In addition, the results showed that adults with DD properly rated their numerical skills. In fact, adults with DD scored significantly lower than control participants in the NADL Interview (which inquires about the individual’s perception of their ability to deal with the numerical activities of everyday life), suggesting that they are fully aware of their numerical difficulties. Acknowledging difficulties in numerical activities in everyday life involves other metacognitive and emotional capacities outside the numerical domain [42]. Unawareness of deteriorated numerical skills, which was observed in other clinical populations assessed with the NADL instrument [30,32], can exacerbate the consequences of the deficit and influence the compliance of patients when interventions are proposed. The present findings suggest that, although no formal diagnosis or clinical assessment of the deficit was made during their childhood, the participants with DD were generally aware of their numerical deficits in adulthood.

It is worth noting that most of our DD participants were students or workers who requested a clinical assessment in order to benefit from the compensatory and dispensatory tools prescribed by the Italian Law n. 170/2010 regarding specific learning disorders. Many of them were attending or had completed high school and had a good level of education. Therefore, this was a selected group of people with DD, possibly not comparable to the broader category “low numeracy” reported in cohort studies. Our sample, indeed, did not include individuals who showed low mathematics achievements for reasons different from DD, who preferred non-academic training, who did not find a job, or who were forced to follow an intermittent employment career with periods of casual work and unemployment. It is an open question how these accumulated educational and occupational individual experiences shape people’s ability to deal with daily numerical activities. We might hypothesize that, for those individuals, Formal and Informal assessments of mathematics might be more challenging than for individuals in our sample, who made an effort to complete a scholastic and professional path. However, a dissociation between formal mathematical abilities and their use in everyday life might be a possibility as well, since compensatory and motivational strategies can develop progressively on the basis of different types of feedback received in real life. This might be the case, for instance, for people with low numeracy who do not suffer from DD and who eventually could profit more efficiently from—for example—long-term memory numerical facts than people with DD. A better understanding of the compensatory mechanisms of number processing in everyday activities is of great relevance at both the personal and the societal levels. Future studies including a more comprehensive and differentiated sample of people with low numeracy and employing the NADL instrument or similar ecologically valid tools, should help to clarify this issue.

Furthermore, most of our DD participants had comorbidities, such as other specific learning disorders (dyslexia, dyscalculia, dysgraphia) or ADHD. We decided not to exclude these participants from our sample because comorbidities are very common among people with DD [11,13]. The most common reported comorbidity was dyslexia (see Supplementary Materials, Spreadsheet S3). This learning disability might have partially affected participants’ scores. In the NADL formal, although there were no parts requiring reading words or presenting word problems, related tasks such as reading and writing mathematical operations or arithmetic symbols might have been affected by dyslexia in the DD group. On the other hand, the risk that participants’ responses in the NADL Interview and NADL informal sections were affected by dyslexia was minimized on task, since all instructions, questions, and answers were orally provided. For this reason, dyslexia may not have had a significant impact on the conclusions of our study, which focused particularly on the results of the NADL Interview and NADL Informal sections. Only one participant had ADHD, but he did not complete neither the NADL interview nor the informal test. In the formal test, he obtained a score higher than the mean for the DD group. Therefore, we can exclude that his condition compromised the results in the test.

Last but not least, a qualitative description of the psychological and clinical history of the patients obtained through personal interviews revealed, in various cases, states of discomfort associated with mathematical failures from as early as primary school age. Clinical reports documented negative emotions of tension, worry, frustration, anger, and psychosomatic symptoms such as stomachache and insomnia, particularly before exams. These are possibly attributable to mathematical anxiety [43]. Some patients explicitly referred to the fact that, when present, these manifestations intensified negative academic outcomes and, in some cases, grounded the need for psychotherapeutic treatments. The patients also highlighted the feelings of frustration and guilt experienced when their difficulties during childhood were ascribed to lack of effort and motivation. Together, these clinical annotations evidence how numerical deficits have a major impact on the social and emotional well-being of individuals with DD and call for increased efforts to design treatments with an integrated approach.

The present study leaves unresolved the exact nature of the numerical deficits experienced by adults with DD in everyday life. One possibility is that these deficits derive directly and exclusively from their numerical difficulties. However, another possibility is that they are the consequence of cognitive deficits associated with low numerical performance [8,44,45]. Unfortunately, only a small portion of the DD group had completed a full neuropsychological battery at the time of the assessment, and we were unable to reach out to other participants to complete the evaluations of specific cognitive functions in the entire group. Future studies correlating the performances in numerical ecological tests and in tests of attention, working memory, executive functions, etc., should contribute to clarifying this issue. For the time being, the present results suggest that numerical deficits in everyday life cannot be attributed to low intelligence performances, as the results did not change when participants with IQ scores lower than the normal range values (*N* = 1) were excluded from the study.

## 5. Conclusions

Numerical difficulties in adults with DD are not limited to numerosity and arithmetic processing in formal settings, but also negatively impact the individual’s ability to deal with numerical tasks of everyday life. Specifically, the study revealed that individuals with DD are impaired when facing real-world numerical problems of time and measure estimation and experience difficulties when managing simple monetary transactions. In contrast, no evidence of impairments in dealing with distance estimation was observed. Our study also revealed that adults with DD are perfectly aware of their numerical difficulties, which are often related to emotional problems and negatively impact their academic and occupational careers. These results can provide new information for the formulation of interventions for adults with DD. Many efforts have been made in the last decades to support students with mathematical learning difficulties [46,47], but attempts to propose age-appropriate trainings for adults with numerical difficulties are rare [48,49]. Our study highlights the need to design innovative interventions that may target deficiencies in the cognitive, numerical, social, and emotional dimensions of adults with DD.

## Figures and Tables

**Table 1:** Performances of people with DD and controls in the NADL battery.

Test	Maximum Score	DD		Controls		U or Chi Square	P (One-Sided)	r (Effect Size)	Bayes Factor (Rouder Method)
		Mean	SD	Mean	SD				
INTERVIEW *	10	7.82	1.59	9.73	0.45	89.5	<0.001	−0.631	0.000
Time *	5	4.24	1.22	4.85	0.46	175.5	0.004	−0.389	0.444
Measure *	1	0.76	0.44	1.00	0.00	6.93	0.004	−0.384	0.179
Distance	1	0.67	0.48	0.73	0.45	0.228	0.315	−0.069	4.165
Communication	1	0.95	0.22	1.00	0.00	1.26	0.130	−0.164	2.650
General knowledge	7	6.00	0.84	6.42	0.64	196.0	0.038	−0.259	0.874
Money *	8	6.95	0.92	7.62	0.75	156.5	0.003	−0.403	0.208
TOTAL INFORMAL *	23	19.57	2.86	21.62	1.10	130.5	0.001	−0.458	0.047
Total number comprehension	17	16.58	0.76	16.69	0.62	313.0	0.278	−0.082	4.100
Total transcoding	10	9.92	0.28	9.81	0.49	289.0	0.214	−0.112	3.167
Total mental calculation*	18	15.15	1.67	17.23	1.14	102.5	<0.001	−0.614	.000
Total rules *	15	13.5	1.56	14.53	0.71	199.5	0.003	−0.379	0.087
Total written calculation *	17	12.11	2.80	14.81	1.52	142.5	<0.001	−0.500	0.003
TOTAL FORMAL *	77	67.21	4.11	73.07	2.71	71	<0.001	−0.665	0.000

Note. Asterisks indicate significant differences between the groups after Holm–Bonferroni correction for multiple comparisons, *p* ≤ 0.004.

## Data Availability

All data presented in this study are available via the supplementary materials.

## Supplementary Materials

The following supporting information can be downloaded at: https://www.mdpi.com/article/10.3390/brainsci12030373/s1, Spreadsheet S1: Demographic characteristics of the participants; Spreadsheet S2: Raw NADL scores; Spreadsheet S3: Neuropsychological assessment and treatment; Table S1: Comparison between DD and controls for the complete NADL battery.
