# Supplementary material for: Dyscalculia in Early Adulthood: Implications for Numerical Activities of Daily Living
Source: Brain Sci. 2022 Mar 11;12(3):373. doi: 10.3390/brainsci12030373 (PMC8946289; doi:10.3390/brainsci12030373)
Supplement: Supplementary file 1 [file brainsci-12-00373-s001.zip › Table S4 - Comparison between DD and controls for the complete NADL battery.pdf]

Table S4. Performances of people with DD and controls in the complete NADL battery.

TEST	DD Mean	DD SD	Controls Mean	Controls SD	U or Chi square	p (one-tailed)	r (effect size)	Bayes factor (Rouder Method)
INTERVIEW	7.82	1.59	9.73	0.45	89.5	<.001	-0.631	.000
Time	4.24	1.22	4.85	0.46	175.5	0.004	-0.389	.444
Measure	0.76	0.44	1.00	0.00	6.93	0.004	-0.384	.179
Distance	0.67	0.48	0.73	0.45	.228	0.315	-0.069	4.165
Communication	0.95	0.22	1.00	0.00	1.26	0.130	-0.164	2.650
General knowledge	6.00	0.84	6.42	0.64	196.0	0.038	-0.259	.874
Money	6.95	0.92	7.62	0.75	156.5	0.003	-0.403	.208
TOTAL INFORMAL	19.57	2.86	21.62	1.10	130.5	0.001	-0.458	.047
Comparison	5.81	0.40	6.00	0.00	273.0	0.010	-0.323	.373
Number line	0.92	0.27	1.00	0.00	312.0	0.076	-0.198	1.915
Digits	9.85	0.37	9.69	0.62	308.0	0.211	-0.111	2.835
Total number comprehension	16.58	0.76	16.69	0.62	313.0	0.278	-0.082	4.100
Reading numbers	5.00	0.00	4.88	0.43	288.0	0.085	-0.194	2.218
Dictation	4.92	0.28	4.92	0.27	310.0	0.467	-0.012	4.726
Total transcoding	9.92	0.28	9.81	0.49	289.0	0.214	-0.112	3.167
Mental addition	5.69	0.68	5.77	0.59	315.0	0.270	-0.085	4.429
Mental subtraction	5.62	0.57	5.96	0.20	233.5	0.002	-0.388	.132
Mental multiplication	3.85	1.29	5.50	0.71	103.5	<.001	-0.618	.000
Total mental calculation	15.15	1.67	17.23	1.14	102.5	<.001	-0.614	.000
Arithmetical Rules	6.62	0.70	6.88	0.33	281.5	0.066	-0.209	1.191
Addition principles	3.50	0.86	3.85	0.37	269.0	0.047	-0.232	1.014

Multiplication principles	3.38	0.75	3.81	0.40	229.0	0.008	-0.333	.312
Total Rules	13.5	1.56	14.53	0.71	199.5	0.003	-0.379	.087
Written additions	5.15	1.16	5.42	0.81	308.0	0.270	-0.085	3.158
Written subtractions	4.35	1.26	5.50	0.58	168.5	<.001	-0.453	.004
Written multiplications	2.62	1.33	3.88	1.14	162.5	<.001	-0.456	.019
Total written calculation	12.11	2.80	14.81	1.52	142.5	<.001	-0.500	.003
TOTAL FORMAL	67.21	4.11	73.07	2.71	71	<.001	-0.665	.000
